# Circulating Adipocytokines and Chronic Kidney Disease

**DOI:** 10.1371/journal.pone.0076902

**Published:** 2013-10-07

**Authors:** Katherine T. Mills, L. Lee Hamm, A. Brent Alper, Chad Miller, Alhakam Hudaihed, Saravanan Balamuthusamy, Chung-Shiuan Chen, Yanxi Liu, Joseph Tarsia, Nader Rifai, Myra Kleinpeter, Jiang He, Jing Chen

**Affiliations:** 1 Department of Epidemiology, Tulane University School of Public Health and Tropical Medicine, New Orleans, Louisiana, United States of America; 2 Department of Medicine, Tulane University School of Medicine, New Orleans, Louisiana, United States of America; 3 Tulane University Hypertension and Renal Center of Excellence, Tulane University School of Medicine, New Orleans, Louisiana, United States of America; 4 Department of Laboratory Medicine, Children' Hospital, Boston, Massachusetts, United States of America; 5 King Abdulaziz University, Jeddah, Saudi Arabia; University of Leicester, United Kingdom

## Abstract

**Background:**

Adipokines have been associated with atherosclerotic heart disease, which shares many common risk factors with chronic kidney disease (CKD), but their relationship with CKD has not been well characterized.

**Methods:**

We investigated the association of plasma leptin, resistin and adiponectin with CKD in 201 patients with CKD and 201 controls without. CKD was defined as estimated glomerular filtration rate (eGFR) <60 mL/min/1.73 m^2^ or presence of albuminuria. Quantile regression and logistic regression models were used to examine the association between adipokines and CKD adjusting for multiple confounding factors.

**Results:**

Compared to controls, adjusted median leptin (38.2 vs. 17.2 ng/mL, p<0.0001) and adjusted mean resistin (16.2 vs 9.0 ng/mL, p<0.0001) were significantly higher in CKD cases. The multiple-adjusted odds ratio (95% confidence interval) of CKD comparing the highest tertile to the lower two tertiles was 2.3 (1.1, 4.9) for leptin and 12.7 (6.5, 24.6) for resistin. Median adiponectin was not significantly different in cases and controls, but the odds ratio comparing the highest tertile to the lower two tertiles was significant (1.9; 95% CI, 1.1, 3.6). In addition, higher leptin, resistin, and adiponectin were independently associated with lower eGFR and higher urinary albumin levels.

**Conclusions:**

These findings suggest that adipocytokines are independently and significantly associated with the risk and severity of CKD. Longitudinal studies are warranted to evaluate the prospective relationship of adipocytokines to the development and progression of CKD.

## Introduction

The prevalence of chronic kidney disease (CKD) is high and increasing in the US general population [1], [2]. CKD is associated with an increased risk of end-stage kidney disease (ESKD), cardiovascular disease (CVD), and premature death [3–5. CKD and CVD are closely associated with each other and share many common risk factors [6], [7]. Recent research has revealed that adipokines are associated with atherosclerotic CVD [8], but their associations with CKD have not been well characterized.

An elevated level of leptin has been found in patients with ESKD [9], [10], but this association is inconsistent among patients with pre-dialysis CKD: an elevated level in CKD patients [11]–[14] or no difference [15] have been reported. Previous studies have also reported a higher level of resistin among patients with stage 3 and 4 CKD [16]–[18]. In addition, higher adiponectin levels in those with CKD [19] and ESKD [20] or no difference in adiponectin levels between individuals with and without CKD have been reported [13], [17], [21].

Much of the previous work on the association between leptin, resistin, and adiponectin and CKD has produced conflicting results and has originated primarily from studies with small sample sizes and/or unadjusted comparisons between CKD patients and those without CKD. Our objective is to investigate the association of plasma leptin, resistin, and adiponectin with CKD in a large case-control study after adjusting for multiple important risk factors for CKD. Additionally, we will examine the relationship between plasma leptin, resistin, and total adiponectin and severity of CKD, measured by estimated glomerular filtration rate (eGFR) and urinary albumin.

## Subjects and Methods

### Ethics statement

Tulane University Internal Review Board approved this study, and written informed consent was obtained at the screening visit from all study participants. This study adheres to the Declaration of Helsinki.

### Study participants

We recruited 201 CKD patients and 201 controls without CKD in the greater New Orleans, Louisiana area from 2007 to 2010. CKD patients aged 21–74 years were identified from nephrology and internal medicine clinics in the study area. CKD was defined as estimated glomerular filtration rate (eGFR) <60 ml/min/1.73 m^2^ or presence of albuminuria (≥30 mg/24-hours). Patients were excluded if they had a history of chronic dialysis, kidney transplants, uremia, autoimmune or chronic systemic inflammatory diseases, immunotherapy in the past six months, chemotherapy within the past two years, and current clinical trial participation that may have an impact on CKD. Additional exclusion criteria for both cases and controls were history of HIV or AIDS and inability or unwillingness to give informed consent. Controls, defined as having no evidence of CKD (eGFR >60 ml/min/1.73 m^2^ and no persistent albuminuria), were recruited through mass mailing to residents aged 21–74 years living in the same area.

### Measurements

A standard questionnaire was administered by trained staff at a clinical visit to obtain demographic information, lifestyle risk factors (including cigarette smoking, alcohol drinking, and physical activity), self-reported history of CVD, diabetes, hypercholesterolemia, and hypertension, as well as medication use.

Three blood pressure (BP) measurements were obtained at a clinical visit by trained and certified staff using a standard mercury sphygmomanometer according to a common protocol adapted from procedures recommended by the American Heart Association [22]. Body height and weight were obtained by trained staff and used to calculate body mass index (BMI = weight in kg/height^2^ in m).

An overnight fasting blood sample was collected to measure plasma leptin, resistin, adiponectin, and glucose, serum creatinine and cholesterol, and triglycerides. eGFR was estimated from serum creatinine (SCr), sex, age, and race using the CKD-Epi equation: eGFR = 141× min(SCr/κ, 1)^α^× max(SCr/κ, 1)^−1.209^×0.993^Age^×1.018 (if female) ×1.159 (if black), where κ is 0.7 for females and 0.9 for males and α is −0.329 and −0.411, respectively [23]. A 24-hour urinary sample was collected to measure albumin.

Serum cholesterol and triglyceride levels were assayed using an enzymatic procedure on the Hitachi 902 automatic analyzer (Roche Diagnostics, Indianapolis, IN, USA). Serum glucose was measured using a hexokinase enzymatic method (Roche Diagnostics, Indianapolis, IN, USA). Serum creatinine was measured using the Roche enzymatic method (Roche–Hitachi P-Module instrument with Roche Creatininase Plus assay, Hoffman-La Roche, Basel, Switzerland). Urinary concentrations of albumin were measured with a DCA 2000 Analyzer (Bayer AG, Leverkusen, Germany). Plasma leptin and resistin were measured using an enzymatically amplified “two-step” sandwich-type immunoassay (R&D Systems, Minneapolis, MN, USA), and plasma adiponectin was measured using a quantitative sandwich enzyme immunoassay technique (ALPCO Diagnostics Inc., Salem, NH).

### Statistical analysis

Medians and interquartile ranges (IQR) for leptin (ng/mL) and total adiponectin (µg/mL) were calculated for CKD patients and controls, and the Mann-Whitney test was used to test differences in the unadjusted medians. Quantile regression was used to obtain adjusted medians and IQR, and the Wald test was used to test differences in the adjusted medians between CKD patients and controls. These analyses were adjusted for age, gender, race, high-school education, current cigarette smoking, weekly alcohol consumption, regular physical activity (≥ moderate or heavy activities twice per week), BMI, low-density cholesterol (LDL) cholesterol, systolic blood pressure, fasting plasma glucose, and history of cardiovascular disease. All analyses were also conducted adjusting from waist circumference instead of BMI to assess if a particular measure of adiposity has an impact on the results. Means and 95% confidence intervals (CI) were calculated for resistin (ng/mL), a student t-test was used to test for differences in unadjusted means, and ANOVA was used for adjusted means.

Multivariate logistic regression was used to obtain adjusted odds ratios comparing the highest tertile of leptin, resistin, and total adiponectin to the lower two tertiles between CKD patients and controls. Tertiles were defined based upon measurements in the control group [24]. Multivariable linear regression was used to examine the association of eGFR and urinary albumin with leptin, resistin, and total adiponectin, after adjustment for the previously mentioned covariates. Log transformations were used for leptin and total adiponectin, because they are not normally distributed. Multivariate-adjusted regression coefficients are reported associated with a one standard deviation increase in leptin, resistin and adiponectin. All analyses were performed using SAS version 9.2 statistical software (Cary, NC).

## Results

General characteristics of study participants by CKD status are presented in Table 1. Those with CKD were older, less educated, heavier, and less likely to drink alcohol compared to those without CKD. In addition, they were more likely to have a history of CVD, hypertension, diabetes, and hypercholesterolemia and use lipid lowering, anti-diabetes and antihypertensive medications, as well as aspirin. Systolic BP, plasma glucose, and urinary albumin were significantly higher, while LDL-cholesterol, HDL-cholesterol, and eGFR were lower in CKD patients compared to controls.

**Table 1:** Characteristics of 201 patients with chronic kidney disease and 201 controls.

	CKD patients	Non-CKD controls	
Variables	(n = 201)	(n = 201)	p-value for difference
Age, years	55.9±9.9	52.5±10.0	0.0007
Male, %	55.2	45.3	0.05
Blacks, %	60.7	51.2	0.06
High school education, %	58.5	81.6	<0.0001
Current cigarette smoking, %	53.7	48.8	0.32
Weekly alcohol drinking, %	27.9	59.2	<0.0001
Regular physical activity*, %	53.0	72.9	<0.0001
History of CVD, %	43.7	7.0	<0.0001
History of hypertension, %	88.1	23.9	<0.0001
History of diabetes, %	49.3	5.5	<0.0001
History of hypercholesterolemia, %	65.7	30.9	<0.0001
Lipid-lowering Medication Use, %	23.0	9.0	0.0001
Anti-diabetic Medication Use, %	37.2	3.0	<0.0001
Antihypertensive Medication Use, %	82.7	15.4	<0.0001
Aspirin Use, %	35.6	8.5	<0.0001
Body-mass index, kg/m^2^	32.2±7.8	28.9±6.4	<0.0001
Systolic blood pressure, mmHg	132.2±21.0	122.0±14.7	<0.0001
Diastolic blood pressure, mmHg	77.2±13.5	77.6±9.4	0.77
Plasma glucose, mg/dL	119.9±46.7	103.4±35.4	<0.0001
LDL-cholesterol, mg/dL	103.3±45.9	118.2±30.2	<0.0001
HDL-cholesterol, mg/dL	50.3±15.6	57.7±18.0	<0.0001
eGFR, mL/min/1.73 m^2^	43.3±19.9	96.7±16.8	<0.0001
Urinary albumin, mg/24-hrs	74.5 (12.3, 417.4)	5.9 (4.1, 11.4)	<0.0001

Mean ± standard deviation, percentage, or median (inter-quartile range). LDL =  low-density lipoprotein; HDL =  high-density lipoprotein; eGFR =  estimated-glomerular filtration rate; and CVD =  cardiovascular disease.

* Moderate or heavy physical activity at least twice per week.

Median leptin is significantly higher in cases compared to controls (22.4 ng/mL vs. 11.6 ng/mL; p<0.0001), and remained significantly higher after full adjustment (Table 2). Mean resistin was also higher in cases than in controls, both adjusted and unadjusted (16.2 vs. 8.8 ng/mL; p<0.0001). No significant difference was observed in either unadjusted or fully adjusted median total adiponectin between CKD cases and controls.

**Table 2:** Adipocytokine levels according to chronic kidney disease status.

	Unadjusted median (IQR)			Multivariable-adjusted median (IQR)*		
	CKD patients	Non-CKD controls		CKD patients	Non-CKD controls	
Adipocytokines	(n = 201)	(n = 201)	p-value for difference	(n = 201)	(n = 201)	p-value for difference
Leptin (ng/mL)	22.4	11.6	<0.0001	38.2	17.2	<0.0001
	(10.8, 58.9)	(4.7, 25.0)		(24.1, 56.4)	(11.7, 24.5)	
Resistin (ng/mL) †	16.2	8.8	<0.0001	16.2	9.0	<0.0001
	(15.1, 17.4)	(8.2, 9.5)		(15.2, 17.2)	(7.9, 10.0)	
Total Adiponectin (µg/mL)	5.2	5.1	0.5	5.9	5.5	0.1
	(3.5, 9.4)	(3.4, 8.4)		(3.7, 10.1)	(4.3, 7.7)	

IQR =  inter-quartile range.

* Adjusted for age, gender, race, high school education, current cigarette smoking, weekly alcohol consumption, physical activity, body-mass index, LDL-cholesterol, systolic blood pressure, serum glucose, and history of cardiovascular disease.

† Mean (95% confidence interval).

In logistic regression analyses adjusted for age, gender and race, those in the highest tertile of leptin and resistin are significantly more likely to have CKD compared to the lower two tertiles (Table 3). The highest tertile of total adiponectin has a non-statistically significant increased risk of CKD compared to the two lower tertiles. Additional adjustment for high school education, current cigarette smoking, weekly alcohol consumption, physical activity, LDL-cholesterol, systolic BP, and serum glucose did not substantially change the associations between leptin, resistin, and adiponectin and CKD. After further adjustment for BMI, the highest tertiles of leptin, resistin, and adiponectin were significantly associated with CKD compared to the lower two tertiles. Adjusting for waist circumference instead of BMI did not substantially change the associations (results not shown). In a final model with additional adjustment for history of CVD, the odds of CKD in the highest tertiles of leptin, resistin, and adiponectin remained significantly higher than the odds in the lower two tertiles.

**Table 3:** Odds ratios of chronic kidney disease associated with highest tertile compared to the lowest two tertiles adipocytokines.

	Age, gender, and race-adjusted		Multivariable-adjusted*		Multivariable-adjusted**		Multivariable-adjusted†	
	OR (95% CI)	p-value	OR (95% CI)	p-value	OR (95% CI)	p-value	OR (95% CI)	p-value
Leptin (ng/mL)								
<20.35	1.0 (ref)	<0.0001	1.0 (ref)	0.0002	1.0 (ref)	0.006	1.0 (ref)	0.03
≥20.35	3.89 (2.29, 6.60)		3.20 (1.73, 5.91)		2.74 (1.34, 5.61)		2.32 (1.09, 4.94)	
Resistin (ng/mL)								
<9.42	1.0 (ref)	<0.0001	1.0 (ref)	<0.0001	1.0 (ref)	<0.0001	1.0 (ref)	<0.0001
≥9.42	11.2 (6.8, 18.6)		10.5 (5.9, 18.7)		11.0 (6.1, 20.0)		12.7 (6.5, 24.6)	
Total Adiponectin (µg/mL)								
<7.24	1.0 (ref)	0.1	1.0 (ref)	0.06	1.0 (ref)	0.02	1.0 (ref)	0.03
≥7.24	1.44 (0.91, 2.30)		1.70 (0.98, 2.95)		2.03 (1.14, 3.61)		1.95 (1.06, 3.56)	

* Adjusted for age, gender, race, high school education, current cigarette smoking, weekly alcohol consumption, physical activity, LDL-cholesterol, systolic blood pressure, and serum glucose.

** Additionally adjusted for body mass index.

† Additionally adjusted for history of cardiovascular disease.

Multivariate-adjusted linear regression of leptin, resistin, and adiponectin with eGFR and urinary albumin showed a significant inverse association with eGFR and a significant positive association with albumin and each of the adipocytokines (Table 4). The direction of these associations is consistent with leptin, resistin, and adiponection being positively associated with CKD severity.

**Table 4:** Multivariable-adjusted regression coefficients (95% confidence intervals) of GFR and urinary albumin associated with a one standard deviation difference in adipocytokines.

	eGFR, mL/min/1.73 m^2^		Log (urinary albumin, mg/24-hrs)	
	β (95% CI)	p-value	β (95% CI)	p-value
Log (leptin, 1.25 ng/mL)	−12.7 (−17.4, −7.9)	<0.0001	0.64 (0.34, 0.94)	<0.0001
Resistin (7.58 ng/mL)	−15.0 (−17.7, −12.4)	<0.0001	0.74 (0.56, 0.91)	<0.0001
Log (total adiponectin, 0.66 µg/mL)	−8.7 (−11.7, −5.6)	<0.0001	0.36 (0.16, 0.57)	0.0004

eGFR =  estimated-glomerular filtration rate.

* Adjusted for age, gender, race, high school education, current cigarette smoking, weekly alcohol consumption, physical activity, body-mass index, LDL-cholesterol, systolic blood pressure, serum glucose, and history of cardiovascular disease.

## Discussion

The results of the current study show that plasma leptin and resistin concentrations are significantly higher in patients with CKD compared to those without, while there was no significant difference in median total adiponectin between CKD patients and controls. There was a significant and independent association of elevated levels of leptin, resistin, and adiponectin with odds of CKD. In addition, leptin, resistin, and adiponectin are significantly positively associated with severity of CKD measured by eGFR and urinary albumin. The observed associations are independent of established risk factors for CKD, including systolic blood pressure, serum glucose, history of cardiovascular disease, and body mass index.

Our study has important clinical implications. Our findings provide further evidence that leptin and resistin are significantly higher in CKD compared to non-CKD participants. In addition, our results show that leptin, resistin and adiponectin are not only associated with the risk, but also the severity of CKD, independent of BMI and CVD.

Our positive findings of leptin and resistin with CKD are consistent with prior studies [12]–[14], [16]–[18], [25]. Several studies have found significantly higher levels of leptin and resistin in those with CKD compared to healthy controls with minimal or no multivariate adjustment [12], [13], [16], [17], [25]. A recent analysis by Shankar et al using data from the Third National Health and Nutrition Examination Survey of 5,820 representative adults in the United States found the odds of CKD to be 3.25 (95% CI: 1.61, 6.55) times higher in the highest quartile of leptin compared to the lowest quartile after adjusting for relevant covariates [14]. A study of 3,192 Japanese subjects found an odds ratio for CKD of 2.32 (95% CI: 1.71, 3.16) comparing those in the highest resistin tertile to the lowest [18]. Our case-control study confirms these previous findings after adjustment for relevant covariates and has the additional strength of using proteinuria to define CKD, which was not a part of the case definition in previous multivariate analyses and allows for a more accurate classification of CKD cases and non-cases.

Adiponectin has anti-inflammatory properties [26], [27] and is inversely associate with obesity, coronary artery disease, and type 2 diabetes [26], [28], [29]. Contrary to these findings, prior studies of adiponectin and CKD have shown either higher adiponectin levels in those with CKD compared to those without CKD (19.2 µg/mL vs 14.8 µg/mL; p<0.01) [19] or no difference in adiponectin levels between the two groups in unadjusted analyses [13], [17], [21]. While we observed no difference between the median values of adiponectin between cases and controls, those with higher adiponectin values have greater odds of CKD in our study, and the association remained significant after multivariate adjustment. In addition, we found a significant positive association between adiponectin and markers of disease severity consistent with some prior studies [30]–[31].

This study has several strengths and limitations. First, these analyses had a larger sample size than most previous studies of these associations. Second, these analyses adjusted for many CKD risk factors, so the independent association between adipocytokines and CKD could be assessed. Third, proteinuria was measured in this population and used to define CKD allowing for a more accurate classification of case status than eGFR alone. The cross-sectional nature of this study does not allow us to determine if the higher levels of adipocytokines lead to kidney damage and CKD or if the higher levels are a result of kidney damage associated with CKD. Longitudinal studies are needed to further understand these associations.

In conclusion, our results show that leptin, resistin, and adiponectin are associated with the risk and severity of CKD. These results suggest that longitudinal studies and clinical trials should be conducted to investigate if adipocytokines play a role in the development and progression of CKD independent of BMI or waist circumference. These important findings may advance our further understanding of risk factors for CKD.
